# Novel Endourological technique for a better navigation in incontinent urinary diversion (ileal conduit) during Double J stent removal

**DOI:** 10.1016/j.eucr.2023.102549

**Published:** 2023-08-29

**Authors:** Ricardo Miyaoka, Wilmar Azal Neto, Renato Nardi Pedro

**Affiliations:** a Division of Urology, Faculty of Medical Sciences, State University of Campinas – UNICAMP, Cidade Universitária Zeferino Vaz, Rua Vital Brasil, 80, CEP 13083-888, Campinas, SP, Brazil; b Clínica Urologia Campinas, Av. Francisco Glicério, 2331, salas 63/64, edifício Glicerio Office, Vl. Itapura, CEP 13020-210, Campinas, SP, Brazil

**Keywords:** Radical cystectomy, Ileal conduit, Stent removal, Flexible ureteroscopy, Double J stent

## Abstract

**Introduction:**

Assessing ileal conduit for double J stents removal after radical cystectomy is not always a straightforward task as navigation inside the ileal loop can be challenging to manage due to the difficulty to maintain a waterfilled environment and its long and tortuous aspect.

**Methods:**

We present a novel technique using a flexible ureteroscope that aims to ease this common demand with simple and readily available tools.

**Results:**

This technique has been successfully utilized in 2 patients now. No complications were documented

**Conclusion:**

We propose a novel surgical technique to improve endoscopic navigation in incontinent ileal loop urinary diversion.

## Highlights

• Flexible ureteroscope may be easily used through a cut-end 3-way 24F Foley catheter to facilitate endoscopic navigation into an ileal conduit.
• This technique may specially benefit ureteral stents withdrawal.
• This surgical approach is easily reproducible and based upon ordinary endoscopic equipment.
• Navigation is easier than using a flexible nephroscope.

## 1 Introduction

Bladder cancer is the fourth most prevalent cancer among men in the Western World following prostate, lung and colon cancers^1^; the risk of developing bladder cancer is 2–4% in men and 0,5%–1% in women.^2^

According to guidelines, radical cystectomy is the standard of care for muscle invasive bladder cancer (>T2) and for tumors that cannot be controlled with transurethral resection.^3^ Following the excision of the bladder the most frequent urinary diversion is the Ileal conduit.^4^ Both ureteroileal anastomosis are secured with stents to aid in healing, to prevent leakage and therefore decrease the risk of stenosis. Double J stents have recently been associated with better clinical outcomes when compared to external ureteral catheters.^5^

The posterior removal of double J stents in this population usually requires using a flexible cystoscope; however, it can be challenging due to the sometimes long and tortuous anatomy of the ileal conduit. Moreover, maneuvering the flexible scope into it requires patience and dexterity as the incontinent nature of the conduit doesn't allow saline distension of the loop making it even more time consuming.

The present manuscript describes an original method to facilitate navigation and double J stent removal in a patient with ileal conduit following radical cystectomy that uses readily available urological devices.

## 2 Case presentation

A new endourological technique has been developed to expedite double J stent removal in ileal conduits.

The technique was performed in an elderly 72 years-old male patient who underwent uneventful robotic radical cystectomy with total intracorporeal ileal conduit urinary diversion for treatment of muscle invasive bladder cancer. He had a 6Fr double J stent placed in each ureter before completion of ureteroileal anastomosis, and stent removal was carried out 8 weeks after initial surgical procedure.

### 2.1 Endourological technique description

**Fig. 1:**
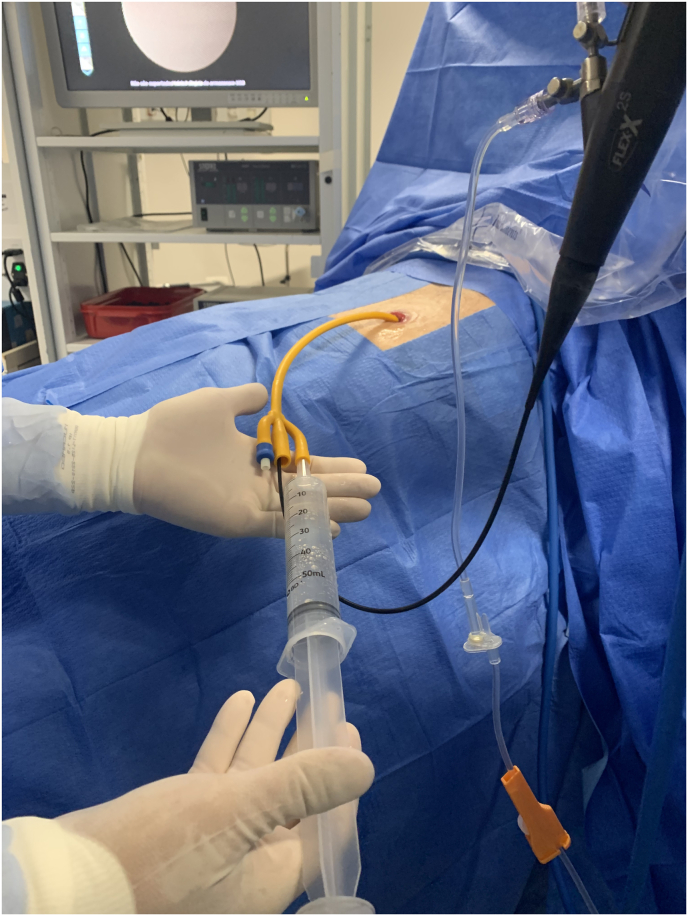
Foley catheter is introduced into ileal conduit stoma and ureteroscope is inserted throught it (cut-end). Third way is used to liquid fill the conduit.

**Fig. 2:**
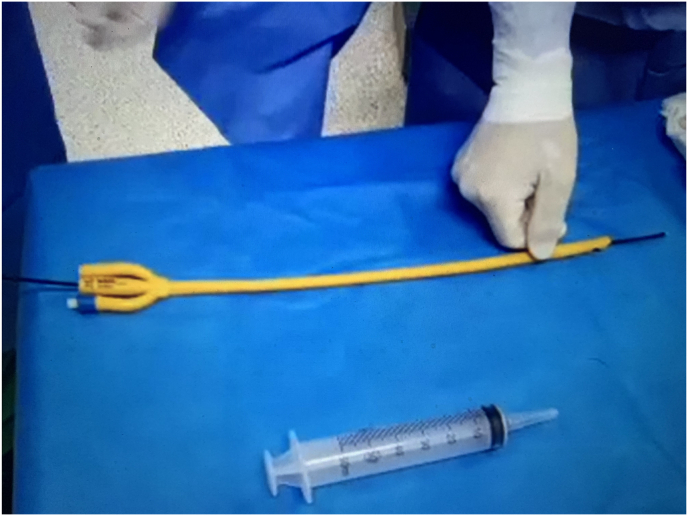
7.5F Flexible ureteroscope is easily passed through cut-end 24F 3-way Foley catheter.

**Fig. 3:**
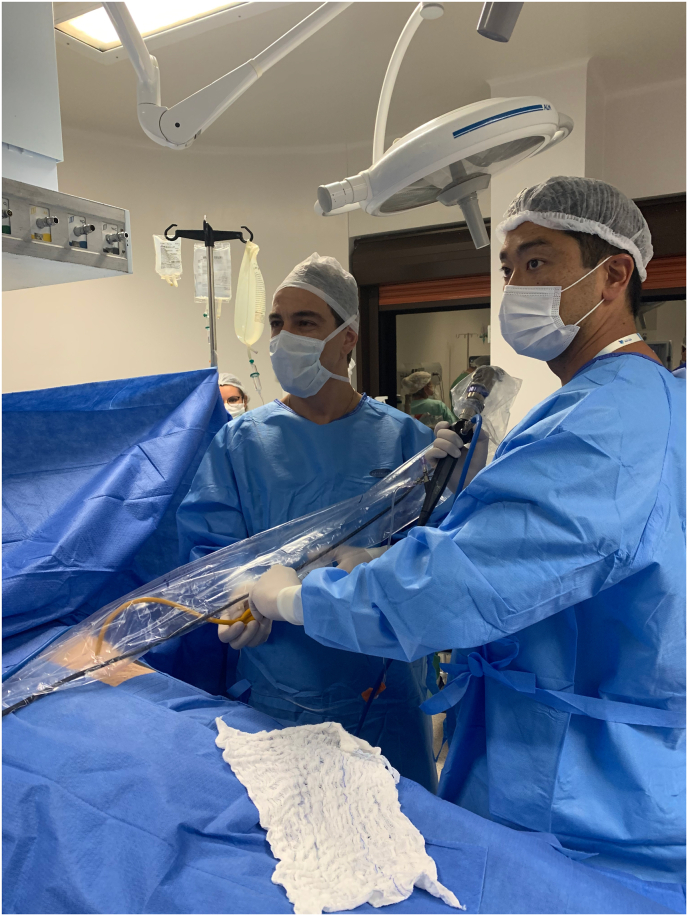
Detail of assistant's help in keeping catheter inside the stoma, stable and straight to facilitate scope insertion, irrigation and visualization.

**Fig. 4:**
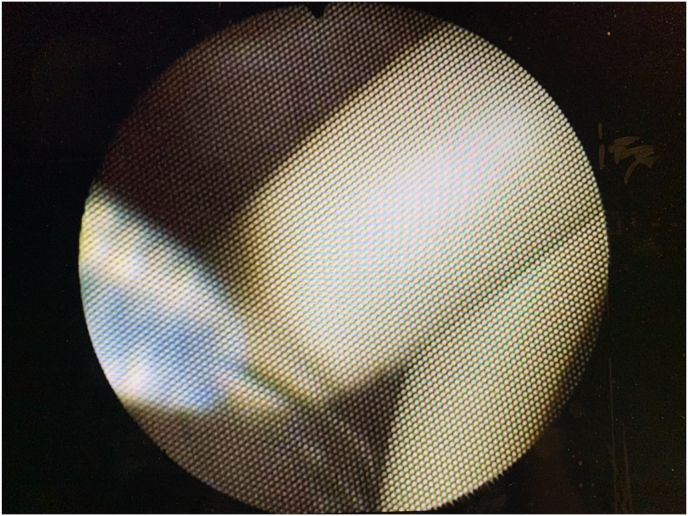
Endoscopic view of double J lower end inside the ileal loop and extractor basket (on the left) ready to open and grasp the tip of the stent (on the right).

**Fig. 5:**
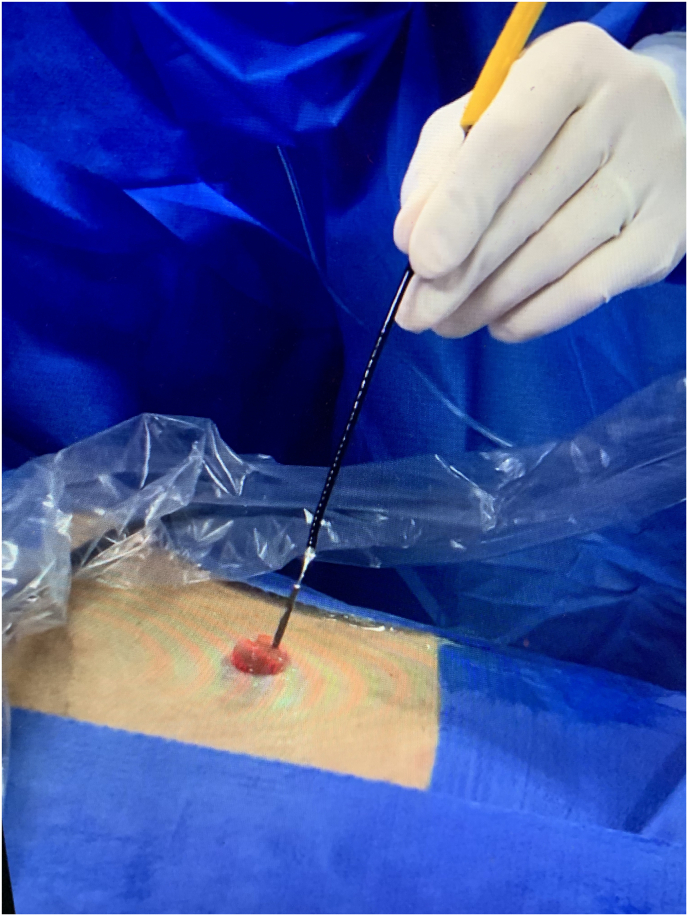
Stent final removal after grasping the ureteral stent end and ureteroscope is pulled out along with the basket.

Patient was positioned in dorsal decubitus under general anesthesia and proper surgical prep was done. A 24Fr 3-way Indwelling Foley catheter was carefully cut off at the very distal tip with care not to damage the balloon. The catheter was then introduced into the stoma until the balloon was completely inside the conduit, then it was inflated with up to 5 mL of distilled water to keep the catheter in place and to avoid liquid backflow around the catheter. The ileal conduit was then filled with saline through the irrigation channel of the catheter by using a IV tubing set or a 60mL syringe (Fig. 1). Filling the Ileal conduit with saline not only provided an adequate environment for endoscopic navigation but also helped to aligning the often-tortuous ileal conduit. A 7.5F flexible fiber optic ureteroscope (STORZ Flex X^2^) was then inserted through the main opening of the 24F Foley catheter while keeping the 3rd way closed after conduit filling. The ureteroscope could be smoothly inserted through the drainage channel of the Foley catheter with no resistance (Fig. 2). It is highly advisable to have a surgical assistant to help keeping the Foley catheter in place and as straight as possible to facilitate ureteroscope insertion (Fig. 3). Endoscopic navigation throughout the conduit was performed without difficulties and the stents were then easily visualized at the bottom of the loop (Fig. 4). Stent end was grasped with an ordinary 1.5–2.2 nitinol tipless stone extractor (N-Compass Cook Medical) and pulled out from the stoma (Fig. 5). Patient was discharged from the hospital after full general anesthesia recovery.

Procedure was concluded in less than 40 minutes with no complications.

## 3 Discussion

The most common urinary diversion following radical cystectomy is the ileal conduit for its expeditious execution even if performed fully intracorporeally in robot assisted laparoscopic approach. In 1983, Jarowenko and Bennet described the use of a single J stent in order to avoid stricture formation^6^. It is well known the advantages of placing a ureteral stent through the ureteroenteric anastomosis to aid the healing process during periods of up to 8 weeks of time.^7^ The Double J stent has been associated with better clinical outcomes and it has been a popular option amongst urologists; however, attention should be paid not to postpone stent withdraw due to risks of stent encrustation, urinary infection and even sepsis specially in this group of patients.^8^ Thus, it is important to schedule ureteral stent removal.

When using single J catheter, the distal end of the 90 cm long stent comes out of the ileal loop allowing its removal by simple manual traction.^6^ At times, standard double J stents are used. Although ileal peristalsis may spontaneously expel the stents after a few weeks, this may not occur and active removal may be needed.

Removing the double J stents from an ileal conduit can be tricky and time-consuming as the intestinal loop is often tortuous and long, making it difficult to navigate with a flexible cystoscope. In an attempt of improving endoscopic maneuverability throughout the ileal conduit the use of a gastroscope or a duodenoscope has been reported.^9^^,^^10^ Also, an extended double J catheter was designed by Klutke et al.^11^ aiming to facilitate stent replacement or removal. However, the fabrication of this device was discontinued.

Our technique offers the advantage of using a common urological device, readily available for most urologists worldwide; therefore, there is no need to use instruments urologists are not familiar with and it does not add costs to the procedure. The idea of using a cut-end standard Foley catheter has been previously described for diagnostic purposes such as to delineate the ileal conduit anatomy, known as loopogram.^12^ To our knowledge, this is the first report on using a 3-way Foley catheter for ileal conduit flexible endoscopy as an “access sheath” and as a provider of watertight filling device.

## 4 Conclusion

Double J stent removal from ileal conduits can be expedite with a new and practical endourological technique. Moreover, the present technique may facilitate navigation with flexible ureteroscope for diagnostic and therapeutic procedures in patients with ileal conduits.

## Declaration of competing interest

None.
